# Nitrogen and carbon isotope variability in the green-algal lichen *Xanthoria parietina* and their implications on mycobiont–photobiont interactions

**DOI:** 10.1002/ece3.417

**Published:** 2012-11-08

**Authors:** Andreas Beck, Christoph Mayr

**Affiliations:** 1 Lichenology and Bryology Department, Botanische Staatssammlung MünchenMenzinger Strasse 67, D–80638, München, Germany; 2 GeoBio-Center, Ludwig-Maximilians Universität MünchenRichard-Wagner-Str. 10, D–80333, München, Germany; 3 Department für Geo- und Umweltwissenschaften, Ludwig-Maximilians Universität MünchenRichard-Wagner-Str. 10, D–80333, München, Germany

**Keywords:** Lichen symbiosis, mycobiont, photobiont, stable isotope, substrate, *Xanthoria parietina*, δ^13^C, δ^15^N

## Abstract

Stable isotope patterns in lichens are known to vary largely, but effects of substrate on carbon and nitrogen stable isotope signatures of lichens were previously not investigated systematically. N and C contents and stable isotope (δ^15^N, δ^13^C) patterns have been measured in 92 lichen specimens of *Xanthoria parietina* from southern Bavaria growing on different substrates (bark and stone). Photobiont and mycobiont were isolated from selected populations and isotopically analyzed. Molecular investigations of the internal transcribed spacer of the nuclear ribosomal DNA (ITS nrDNA) region have been conducted on a subset of the specimens of *X. parietina*. Phylogenetic analysis showed no correlation between the symbionts *X. parietina* and *Trebouxia decolorans* and the substrate, isotope composition, or geographic origin. Instead specimens grown on organic substrate significantly differ in isotope values from those on minerogenic substrate. This study documents that the lichens growing on bark use additional or different N sources than the lichens growing on stone. δ^15^N variation of *X. parietina* apparently is controlled predominantly by the mass fraction of the mycobiont and its nitrogen isotope composition. In contrast with mycobionts, photobionts of *X. parietina* are much more ^15^N-depleted and show less isotopic variability than mycobionts, probably indicating a mycobiont-independent nitrogen acquisition by uptake of atmospheric ammonia.

## Introduction

Lichen growth is most notable on substrate, where almost no other organisms can grow, particularly due to low or absent water storage in the environment or where some factor like low temperature excludes higher plants (e.g., Garvie et al. 2008). Frequently lichens colonize nutrient-poor substrates, and especially N-limitation seems to be a considerable limiting factor (Crittenden et al. 1994; Nash 2008). In the last decades, the use of stable isotope techniques has become an important tool for ecophysiology and ecosystem research (e.g., Högberg 1997; Dawson et al. 2002; Post 2002), but was comparatively rarely applied to lichens. Krouse (1977) was among the first conducting stable isotope measurements on lichens and elucidated the uptake of atmospheric sulfur emissions using their δ^34^S values. Since then δ^34^S values were repeatedly used to study the response of lichens to atmospheric sulfur pollution (e.g., Wiseman and Wadleigh 2002).

The δ^13^C and δ^15^N values of lichens were in the focus of comparatively few studies so far. In general, δ^13^C values of lichens were found to vary broadly over a large range of habitats and species (e.g., Batts et al. 2004), but δ^13^C values of lichens appeared to be less variable within one lichen species, for example, *Usnea antarctica* Du Rietz, when growing in less diverse habitats not influenced by anthropogenic emissions, for example, Antarctic islands (Galimov 2000). As all lichen photobionts use the standard Rubisco (Máguas et al. 1995), the most notable influence on the δ^13^C value of lichens is the δ^13^C of the CO_2_ source and diffusion resistance, where the ratio of internal to external CO_2_ determines the final discrimination (Farquhar et al. 1989; Lakatos et al. 2007; Marshall et al. 2007). Consequently, factors influencing diffusion resistance will have significant effects on the δ^13^C value. One such factor is water content (e.g., Lange et al. 1988) and more positive δ^13^C values in plants are generally used as indicator for desiccation stress (e.g., epiphytes on thin vs. thick branches; Hietz et al. 2002). But this cannot be easily adopted to lichens due to different diffusion pathways of water vapor and CO_2_ in lichen thalli as opposed to higher plants, where both occur via the stomata. Nevertheless, more positive δ^13^C values in drier habitats have also been found for the lichens *Ramalina celastri* (Spreng.) Krog & Swinscow and *R. subfraxinea* Nyl. by Krouse & Herbert (1996, cited in Batts et al. 2004) and for *Cladonia aggregata* (Sw.) Nyl. by Batts et al. (2004), as well as for lichens collected in summer in France by Riera (2005). In contrast, Cuna et al. (2007) found a weak positive correlation between δ^13^C values of lichens (*Cladonia fimbriata* (L.) Fr. and *Pseudevernia furfuracea* (L.) Zopf) and monthly precipitation sums, but a negative correlation with relative humidity. Next to water availability the photosynthetic rate of lichens is limited by light (Palmqvist 2000; Lange et al. 2001) and consequently light levels influence photosynthesis and hence alter the CO_2_ gradient inside the lichen thallus as well.

Lichens utilize different N sources that may be distinguished by their isotopic fingerprints. On the one hand, the negative δ^15^N values observed in epiphytic and mat-forming lichens were explained by the uptake of NH_4_^+^ and NO_3_^−^ from rainwater (Hietz et al. 2002; Ellis et al. 2003; Fogel et al. 2008), as these atmospheric sources have generally negative δ^15^N values (Moore 1977; Heaton 1986; Cornell et al. 1995). On the other hand, several lichen taxa have cyanobacterial photobionts and are known to be able to fix substantial amounts of atmospheric N_2_ (Forman 1975; Matzek and Vitousek 2003). Atmospheric nitrogen, the standard for presenting nitrogen stable isotope ratios, has a δ^15^N value of 0‰ by definition and N_2_-fixation is one of the few biological processes in the N-cycle with little or no isotopic fractionation (Hoering and Ford 1960; Fogel and Cifuentes 1993; Hübner 1986). Possibly the first record on δ^15^N values of lichens was given by Virginia and Delwiche (1982). These authors reported a positive δ^15^N value for the N_2_-fixing *Lobaria oregana* (Tuck.) Müll. Arg. (+0.8‰) and slightly negative values for the tripartite lichen *L. pulmonaria* (L.) Hoffm., the green-algal lichens *Umbilicaria phaea* Tuck., and *Letharia vulpina* (L.) Hue with values between −0.6 and −2.8‰. δ^15^N values of five tropical montane cloud forest canopy lichens ranged around −8 or −1.5‰, respectively, and were either among the highest or the lowest δ^15^N values and N concentrations of all investigated plants (Hietz et al. 2002). Albeit the lichens were not identified, Hietz et al. (2002) speculated that lichens with high N content and δ^15^N values closer to 0‰ can be attributed to N_2_-fixers. Nadelhoffer et al. (1996) reported δ^15^N values between −3 and 1.5‰ for unidentified lichens in a tundra ecosystem. A much wider δ^15^N range (−2.6 to −12.4‰) was found in unidentified lichens from Iceland (Wang and Wooller 2006). The reasons for lichen δ^15^N variability in most studies remain unexplained what is expected as more than 10 processes can alter δ^15^N values and these can hardly be separated in field studies (see Robinson 2001 for a review).

**Figure 1:**
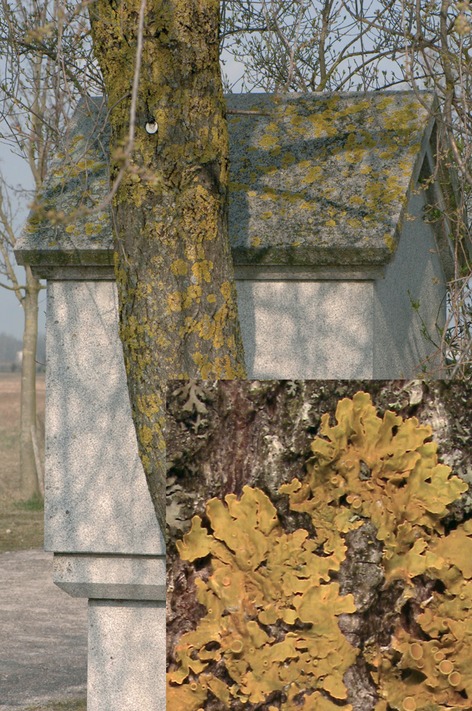
*Xanthoria parietina* growing at one of the sites of investigation (Oberschleißheim near Munich). Inset shows a close-up of the thalli (dimensions correspond to 2.5 × 2.7 cm in reality).

The morphologically complex foliose thalli of *Xanthoria parietina* (L.) Beltr. (Fig. 1) are the result of a long interaction history between the symbiotic myco- and photobiont. But our understanding of these interactions is still far from complete. While the movement of carbohydrates between lichen symbionts has been investigated rather intensively (for reviews see, e.g., Honegger 1991; Eisenreich et al. 2011), the knowledge on nitrogen (N) movement in lichens is much less. Except for lichens containing cyanobacteria, lichens depend on the deposition of nutrients directly on the thallus (Nash 2008). In tripartite lichens (containing cyanobacteria as well as green algae) like *Peltigera aphthosa* (L.) Willd. about 5% of the N fixed by the cyanobiont and released as ammonia was transferred by the fungus to the photobiont *Coccomyxa*, thus covering large parts of its N requirements (Rai et al. 1981; Rai 1988). For the pathway of nitrogen acquisition by the green-algal photobiont, at least three alternative concepts exist. Crittenden (1989) argues for a nitrogen acquisition mainly from living fungal cells and only a minority through the external fungal tissue, based on the findings in *Peltigera* mentioned above, and the observed nutrient movement in decomposing *Cladonia* mats. On the other hand, Honegger (1991) and Richardson (1999) argue that in foliose green-algal lichens like *Xanthoria*, lichen fungi and algae compete for dissolved nutrients in the apoplast, given the hydrophobic interface – no capillary water is present on the outer side of the hydrophobic wall surfaces of the thalline interior. As a third pathway, a gaseous exchange at the photobiont and mycobiont surface might occur. This would not be negatively affected by any hydrophobins. The analysis of nitrogen isotope ratios of lichen symbionts may shed some light on possible nutrient relationships and competition.

The aim of this study was the investigation of the nitrogen and carbon stable isotope composition of green-algal lichens with respect to intraspecies variability due to different substrates and sites. The isotopic signatures of photobiont and mycobiont have also been investigated separately in order to elucidate their respective contributions to the symbiont's isotope signal and elemental contents. The isotopic results presented give information about nutrient availability, different nitrogen sources used, and symbiotic interactions.

## Materials and Methods

### Sites and sampling

All samples were collected in Bavaria, southern Germany, between 11° 01′ E to 11° 56′ E longitude and 48° 14′ N to 47° 32′ N latitude, detailed in Table 1.

**Table 1:** Details for the collecting localities of the green-algal lichen *Xanthoria parietina* used for isotopic analyses

Locality	Altitude	Coordinates
Oberschleißheim	*ca*. 500 m a.s.l.	11° 35′ E, 48° 14′ N
München, Fasanerie (FA)	*ca*. 525 m a.s.l.	11° 30′ E, 48° 07′ N
München, Landsberger Str. (LS)	*ca*. 525 m a.s.l.	11° 30′ E, 48° 07′ N
München, Westpark (WP)	*ca*. 525 m a.s.l.	11° 30′ E, 48° 07′ N
Planegg	*ca*. 550 m a.s.l.	11° 25′ E, 48° 07′ N
Gauting	*ca*. 580 m a.s.l.	11° 24′ E, 48° 03′ N
Maising	*ca*. 640 m a.s.l.	11° 17′ E, 47° 58′ N
Pähl	*ca*. 600 m a.s.l.	11° 11′ E, 47° 55′ N
Weilheim	*ca*. 560 m a.s.l.	11° 08′ E, 47° 49′ N
Fischbachau	*ca*. 780 m a.s.l.	11° 56′ E, 47° 42′ N
Unterammergau	*ca*. 800 m a.s.l.	11° 01′ E, 47° 36′ N
Farchant	*ca*. 670 m a.s.l.	11° 08′ E, 47° 32′ N

### Taxon analysis with molecular methods

*Xanthoria parietina* belongs to a species group that contains two additional species, namely *X. aureola* (Ach.) Erichsen and *X. calcicola* Oksner. These species differ in their substrate choice, with *X. aureola* growing on (seashore) rocks, *X. calcicola* growing on calcareous rocks and walls (only rarely on tree bark) and *X. parietina* having the widest substrate range growing on all these substrates. As incorrect species assignment might affect a study on the influence of the substrate on stable isotope composition within one species, unequivocal taxa assignment has been considered an important issue. Moreover, genetic data provides important information on intraspecies variability, which likewise adds important information for the analysis of the stable isotope composition. Thus, molecular investigations of the ITS nrDNA region have been conducted on selected samples in addition to careful examination of reliable morphological characters to assure proper species assignment (see Lindblom and Ekman 2005, 2007 for details).

DNA has been isolated using the PCR Template Preparation Kit from Roche (Mannheim, Germany; Cat No. 11 796 828 001) following the manufacturers protocol, but grinding the lichen carefully using a micropestle in liquid nitrogen before adding the lysis buffer. 10–50 mg of air-dried lichen material has been used for the DNA extraction. A volume of 1 μL of a 1:10 dilution of the obtained DNA solution has been used for PCR using algal primers as described by Beck and Koop (2001) and fungal primers as described in Dolnik et al. (2010) but using the newly designed primer ITS4m (GCC GCT TCA CTC GCC GTT AC) instead of ITS4. All PCR products were purified with the Macherey-Nagel columns (Macherey-Nagel, Düren) and labelled with Big Dye Terminator v3.1 Kit (Applied Biosystems, Darmstadt, Germany). Cycle sequencing was 30 cycles of: 95°C for 10 sec, 50°C for 15 sec, 60°C for 3 min. Postsequencing cleanup was performed using gelfiltration with Sephadex G-50 Superfine (GE Healthcare, Uppsala, Sweden; Cat. No. 17-0041-01) following the manufacturer's protocol. Forward and reverse strand sequences were detected in an ABI 3730 48 capillary automatic sequencer (Applied Biosystems) and assembled using the Staden package (http://staden.source forge.net/). Double-stranded sequences were aligned manually jointly with representative sequences obtained from Genbank using Gendoc (http://www.nrbsc.org/downloads/). All sequences used are detailed in Table 2 (photobionts) and Table 3 (mycobionts). The resulting data sets were analyzed under the maximum parsimony (MP) and maximum likelihood (ML) criterion using the program PAUP Version 4.0b10 (Swofford 2000). All characters of the ITS1 and ITS2 region, but not the 5.8S nrDNA, have been included for these calculations. As outgroup, the sequences of *T. asymmetrica* T. Friedl & G. Gärtner (strain SAG 48.88), *T. gigantea* (Hildreth & Ahmadjian) G. Gärtner (strain UTEX 2231), *T. incrustata* Ahmadjian ex G. Gärtner (strain UTEX 784), and *T. showmanii* (Hildreth & Ahmadjian) G. Gärtner (strain UTEX 2234) have been used for the photobionts and sequences of *X. aureola* for the mycobionts. To select the nucleotide substitution model and parameters for the ML searches, a hierarchical likelihood ratio test was carried out as implemented in jModelTest 0.1.1 (Guindon and Gascuel 2003; Posada 2006, 2008). The optimal model was selected under the Akaike information criterion (photobiont data set: TVM+G, −ln: 1835.6953; mycobiont data set: TrN+G, −ln: 958.3756). Heuristic searches have been conducted with 1000 (MP) or 500 (ML) random addition sequence (RAS) replicates, tree bisection reconnection (TBR) branch swapping, Multrees option in effect, saving all trees and collapsing branches with maximum length equal to zero. Statistical support in all trees was assessed by bootstrap analysis (BS; Felsenstein 1985) using 1000 (MP) or 500 (ML) bootstrap replicates with five random-addition sequences per replicate, but multree option not in effect.

**Table 2:** Sequences used in the phylogenetic analysis of photobiont sequences from *Xanthoria parietina*

Taxon	Voucher	Substrate	Locality	GenBank No. ITS
***Trebouxia aggregata***	**UTEX180**			**JF831903**
*T. arboricola*	SAG 219–1a			Z68705
*T. asymmetrica*	SAG 48.88			AJ249565.1
***T. crenulata***	**CCAP219/2**			**JF831904**
*T. decolorans*	UTEX 781			FJ626728.1
*T. gigantea*	UTEX 2231			AF242468.1
*T. incrustata*	UTEX 784			AJ293795.1
*T. showmanii*	UTEX 2234			AF242470.1
***T. decolorans*** **ex** ***Xanthoria parietina***	**M–0102151**	**Bark**	**Germany, Maising**	**JF831923**
***T. decolorans*** **ex** ***X. parietina***	**M–0102152**	**Bark**	**Germany, München**	**JF831914**
***T. decolorans*** **ex** ***X. parietina***	**M–0102304**	**Bark**	**Germany, Pähl**	**JF831921**
***T. decolorans*** **ex** ***X. parietina***	**M–0102304**	**Bark**	**Germany, Pähl**	**JF831922**
***T. decolorans*** **ex** ***X. parietina***	**M–0102305**	**Stone**	**Germany, Pähl**	**JF831919**
***T. decolorans*** **ex** ***X. parietina***	**M–0102305**	**Stone**	**Germany, Pähl**	**JF831920**
***T. decolorans*** **ex** ***X. parietina***	**M–0102305**	**Stone**	**Germany, Pähl**	**JF831905**
***T. decolorans*** **ex** ***X. parietina***	**M–0102306**	**Stone**	**Germany, Pähl**	**JF831915**
***T. decolorans*** **ex** ***X. parietina***	**M–0102307**	**Bark**	**Germany, Pähl**	**JF831917**
***T. decolorans*** **ex** ***X. parietina***	**M–0102307**	**Bark**	**Germany, Pähl**	**JF831906**
***T. decolorans*** **ex** ***X. parietina***	**M–0102308**	**Stone**	**Germany, Fischbachau**	**JF831912**
***T. decolorans*** **ex** ***X. parietina***	**M–0102309**	**Bark**	**Germany, Fischbachau**	**JF831913**
***T. decolorans*** **ex** ***X. parietina***	**M–0102316**	**Stone**	**Germany, Gauting**	**JF831916**
***T. decolorans*** **ex** ***X. parietina***	**M–0102325**	**Bark**	**Germany, München**	**JF831909**
***T. decolorans*** **ex** ***X. parietina***	**M–0102327**	**Stone**	**Germany, Pähl**	**JF831908**
***T. decolorans*** **ex** ***X. parietina***	**M–0102328**	**Bark**	**Germany, Pähl**	**JF831907**
***T. decolorans*** **ex** ***X. parietina***	**M–0102916**	**Bark**	**Germany, Farchant**	**JF831910**
***T. decolorans*** **ex** ***X. parietina***	**M–0102919**	**Stone**	**Germany, Farchant**	**JF831911**
***T. decolorans*** **ex** ***X. parietina***	**M–0102925**	**Bark**	**Germany, Gauting**	**JF831918**

New sequences are marked in bold.

**Table 3:** Sequences used in the phylogenetic analysis of mycobiont sequences from *Xanthoria parietina*

Taxon	Voucher (herbarium)	Substrate	Locality	GenBank No. ITS
*X. aureola* no. 16	2002 Coppins s.n. (E)	Stone	Norway, Hordaland; Sweden, Bohuslän; Scotland, East Lothian	AY438275
*X. aureola* no. 17	2001 Lindblom X188 (priv. herb.)	Stone	Sweden, Skåne	AY438276
*Xanthoria parietina* no. 16	2003 L. Lindblom 14/445 (BG)	Bark	Sweden, Mölle chapel	DQ472227
*X. parietina* no. 15	2003 L. Lindblom 13/400 (BG)	Bark	Sweden, Kullaberg lighthouse	DQ472226
*X. parietina* no. 14	2002 L. Lindblom 10/315 (BG)	Bark	Sweden, Kullaberg, Ransvik	DQ472225
*X. parietina* no. 13	2002 L. Lindblom 9/266 (BG)	Stone	Sweden, Arild, E	DQ472224
*X. parietina* no. 12	2002 L. Lindblom 7/222 (BG)	Bark	Sweden, Hovvalla; Kullaberg lighthouse	DQ472223
*X. parietina* no. 11	2002 L. Lindblom 7/216 (BG)	Bark	Sweden, Hovvalla; Mölle chapel	DQ472222
*X. parietina* no. 10	2002 L. Lindblom 7/212 (BG)	Bark	Sweden, Hovvalla	DQ472221
*X. parietina* no. 9	2002 L. Lindblom 7/211 (BG)	Bark	Sweden, Hovvalla; Kullaberg lighthouse; Mölle chapel	DQ472220
*X. parietina* no. 8	2002 L. Lindblom 7/205 (BG)	Bark	Sweden, Hovvalla	DQ472219
*X. parietina* no. 7	2002 L. Lindblom 7/204 (BG)	Bark	Sweden, Hovvalla; Kullaberg lighthouse	DQ472218
*X. parietina* no. 6	2002 L. Lindblom 7/203 (BG)	Bark	Sweden, Hovvalla; Kullaberg lighthouse; Mölle chapel	DQ472217
*X. parietina* no. 5	2002 L. Lindblom 7/201 (BG)	Bark	Sweden, Hovvalla; Kullaberg lighthouse; Mölle chapel	DQ472216
*X. parietina* no. 4	2002 L. Lindblom 7/199 (BG)	Bark	Sweden, Hovvalla; Kullaberg lighthouse; Mölle chapel	DQ472215
*X. parietina* no. 3	2002 L. Lindblom 7/197 (BG)	Bark	Sweden, Hovvalla; Kullaberg lighthouse; Mölle chapel	DQ472214
*X. parietina* no. 2	2002 L. Lindblom 6/167 (BG)	Bark and Stone	Sweden, Hovs Hallar; Kullaberg lighthouse; Kullaberg, Ransvik; Mölle chapel; Arild, Nabben	DQ472213
*X. parietina* no. 1	2002 L. Lindblom 6/163 (BG)	Bark and Stone	Sweden, Hovs Hallar; Hovvalla; Kullaberg, Ransvik; Mölle chapel; Arild, Nabben; Arild, E	DQ472212
***X. parietina***	**M–0102305**	**Stone**	**Germany, Pähl**	**JF831884**
***X. parietina***	**M–0102307**	**Bark**	**Germany, Pähl**	**JF831885**
***X. parietina***	**M–0102328**	**Bark**	**Germany, Pähl**	**JF831886**
***X. parietina***	**M–0102327**	**Stone**	**Germany, Pähl**	**JF831887**
***X. parietina***	**M–0102325**	**Bark**	**Germany, München**	**JF831888**
***X. parietina***	**M–0102916**	**Bark**	**Germany, Farchant**	**JF831889**
***X. parietina***	**M–0102919**	**Stone**	**Germany, Farchant**	**JF831890**
***X. parietina***	**M–0102308**	**Stone**	**Germany, Fischbachau**	**JF831891**
***X. parietina***	**M–0102309**	**Bark**	**Germany, Fischbachau**	**JF831892**
***X. parietina***	**M–0102306**	**Stone**	**Germany, Pähl**	**JF831893**
***X. parietina***	**M–0102316**	**Stone**	**Germany, Gauting**	**JF831894**
***X. parietina***	**M–0102307**	**Bark**	**Germany, Pähl**	**JF831895**
***X. parietina***	**M–0102925**	**Bark**	**Germany, Gauting**	**JF831896**
***X. parietina***	**M–0102151**	**Bark**	**Germany, Maising**	**JF831897**
***X. parietina***	**M–0102305**	**Stone**	**Germany, Pähl**	**JF831899**
***X. parietina***	**M–0102305**	**Stone**	**Germany, Pähl**	**JF831900**
***X. parietina***	**M–0102304**	**Bark**	**Germany, Pähl**	**JF831901**
***X. parietina***	**M–0102304**	**Bark**	**Germany, Pähl**	**JF831902**
***X. parietina***	**M–0102152**	**Bark**	**Germany, München**	**JF831898**

New sequences are marked in bold.

### Stable isotope analyses

Isotope analyses were performed on 92 samples of lichens and 10 isolated lichen symbiont populations, all collected in Bavaria, southern Germany. Before analysis, external debris was carefully removed from the thalli. All isotopic analyses were performed with a Delta Plus (Thermo-Finnigan, Bremen, Germany) isotope-ratio mass spectrometer coupled with a ConFlo-II interface (Thermo-Finnigan) to an elemental analyzer (NC 2500, Carlo Erba, Italy). Samples have been ground using a mortar and pestle. The resulting powder was combusted at 1080°C with excess oxygen and the sample gases were purified and separated according to standard methods (Mayr et al. 2011). Nitrogen (δ^15^N) and carbon (δ^13^C) stable isotopes are given in the common δ-notation calculated as δ = (*R*_sample_/*R*_standard_ − 1) × 1000 with *R*_sample_ and *R*_standard_ as isotope ratios (*R*) of the heavy isotope to the light isotope of the sample and an international standard, respectively. Samples are reported versus the international standards atmospheric N_2_ (AIR) for nitrogen and Vienna-Pee-Dee Belemnite (V-PDB) for carbon and are given in ‰. Carbon and nitrogen contents of the samples were determined from the peak area of the mass spectrometer analyses weighted by the sample's mass. For calibration, the elemental standards cyclohexanone-2,4-dinitrophenyl-hydrazone (C_12_H_14_N_4_O_4_) and atropine (C_17_H_23_NO_3_) (both Thermo Quest, Rodano, Italy) were used. Statistical tests for the stable isotope measurements were carried out with the software PASW Statistics 17.0 (SPSS, Chicago, IL).

### Isolation of myco- and photobiont

Isolation was conducted as detailed in Fontaniella et al. (2000) for *Evernia prunastri* (L.) Ach. Basically, algal and fungal cells are separated in a sucrose-KI gradient. A total of 200 mg of cleaned, air-dried *X. parietina* (equating material from five lichen thalli) have been homogenized in a high-performance dispenser (Miccra D-8; Fa. Miccra, Germany). After separation, the algal/fungal pellets have been washed three times with 10 mmol/L phosphate buffer pH 7.2. Contamination with the other symbiont was microscopically determined to be less than 3%.

### Mass balance calculations

Concentration-dependent mass balance mixing models (modified from Phillips and Koch 2002) were applied to evaluate the mass percentages (wt%) of photo- and mycobionts in selected lichens. The δ^15^N values and elemental contents of a lichen colony and the photobionts and mycobionts separated from a part of the population were needed for this calculation. The mass fractions f_P_ and f_M_ of photobionts (P) and mycobionts (M), respectively, in the lichen (L) were calculated from the δ^15^N values of P, M, L (δ^15^N_P_, δ^15^N_M_, δ^15^N_L_), and their nitrogen concentrations [N]_P_, [N]_M_ using the following equations:


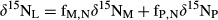
(1)

where δ^15^N_M_, δ^15^N_P_ denotes the isotopic composition of mycobiont and photobiont, respectively, and f_M,N_ and f_P,N_ their fractional nitrogen contributions to total lichen nitrogen. Although the above values can be measured, the fractional biomass contributions of mycobiont (f_M,B_) and photobiont (f_P,B_) have to be calculated with the following equations.

The fractional nitrogen and biomass contributions of photo- and mycobiont sum up to 1:


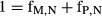
(2)


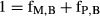
(3)

N contents of myco- and photobiont [N]_M_, [N]_P_ are incorporated in the following equations


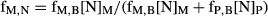
(4)


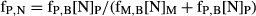
(5)

Substituting equations (4) and (5) in equation (1) gives


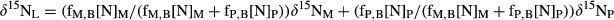
(6)

According to equation (3), f_P,B_ can be substituted by


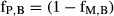
(7)

Thereafter, equation (6) only contained measured variables except of f_M,B_. The optimum value of f_M,B_ was found by iterative fitting so that the calculated δ^15^N_L_ value corresponded to the actually measured value of the total lichen.

## Results

### Relation between substrate and stable isotopes and element contents of *Xanthoria parietina*

**Figure 2:**
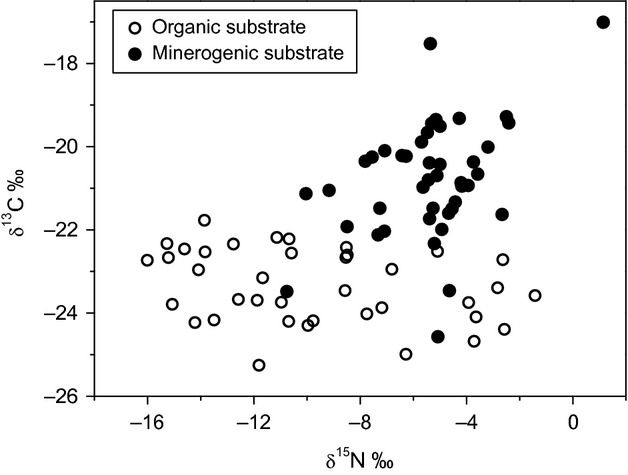
δ^13^C versus δ^15^N values of 82 *Xanthoria* specimens distinguished in those grown on minerogenic (filled dots) and those grown on organic substrate (open circles) from various collection sites in southern Germany.

**Figure 3:**
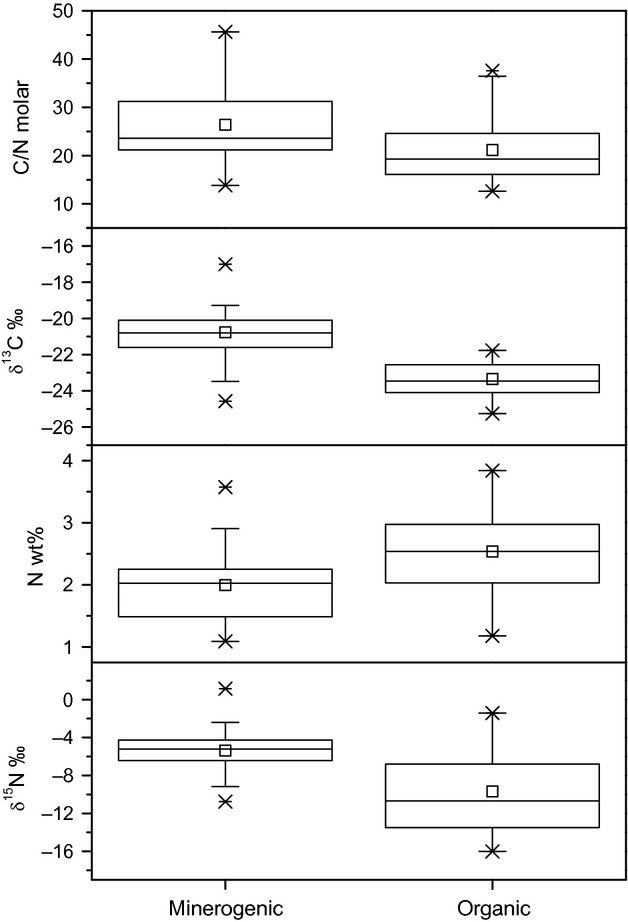
Box-and-whisker plot comparing isotopic compositions, N content, and C/N ratios of *Xanthoria parietina* grown on minerogenic and organic substrate. Whiskers represent the lowest and highest dates within the 1.5 interquartile ranges. Crosses are 1st and 99th percentiles, respectively, and short lines the most extreme values. Means are given by open squares.

The isotope values of *X. parietina* show a large variability ranging from −16.0 to 1.2‰ for δ^15^N and from −17.0 to −25.3‰ for δ^13^C (Fig. 2). δ^13^C values separate the lichens from both substrates. δ^15^N values of bark lichens have a very broad range while those of lichens on minerogenic ground cover only part of that range, but do fall completely within values for bark samples, possibly indicating an extra depleted source for bark lichens that is not available to rock lichens. Specimens that were growing on organic substrate (bark, wood, moss; *N* = 38) have significantly different C/N ratios, N contents, δ^13^C, and δ^15^N values than those grown on minerogenic substrate (concrete, stone, brick, metal; *N* = 44) (Mann–Whitney test, *P* < 0.01; Fig. 3). In contrast, C contents were not significantly different between *Xanthoria* growing on minerogenic versus organic substrates. Thus, higher C/N ratios for specimens growing on minerogenic substrate can be attributed mainly to their lower N contents. *Xanthoria* from minerogenic substrates are on average 4.3 and 2.6‰ enriched in δ^15^N and δ^13^C, respectively, relative to those growing on organic substrates. The range of δ^15^N values for *Xanthoria* from organic substrate is larger compared with those growing on minerogenic ground (Fig. 3).

### Genetic homogeneity of *Xanthoria parietina*

**Figure 4:**
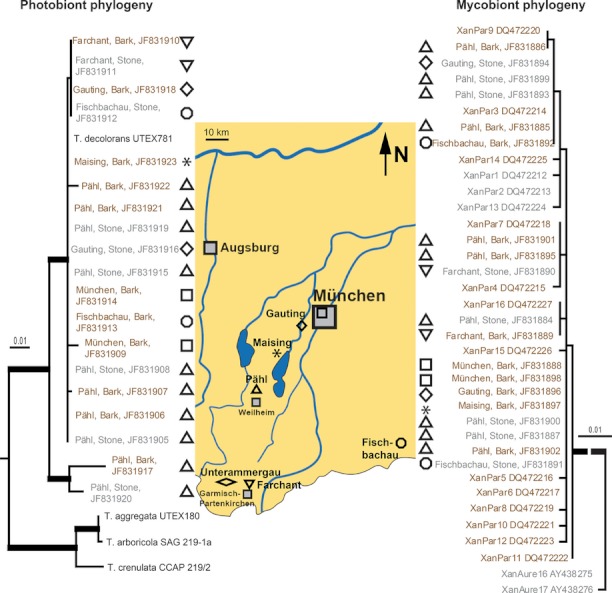
Molecular analysis of the nrITS region of selected specimens of *Xanthoria parietina*. Shown are the single most likely trees of a ML search regarding the nrITS sequences of the photobionts (left-hand side) and mycobionts (right-hand side), respectively, analyzed together with selected sequences from Genbank. A MP search resulted in one tree each with identical tree topology. Branches with high statistical support (≥95% bootstrap support for MP and ≥75% bootstrap support for ML) are indicated by thick lines. For the mycobionts, the line connecting the *X. parietina* sequences has been shortened due to practical reasons, which is indicated by the dashed line. For the photobionts, the distance to the sequences used as outgroup (see Materials and Methods section for details) is not shown. Specimens growing on bark are given in brown color; gray color is used for the specimens from rocks. In the center, a map of southern Bavaria indicates the geographic location of the sampling localities characterized by the respective symbols. Grey filled symbols represent major cities.

In order to secure genetic homogeneity of the investigated material, 19 samples of *X. parietina* have been analyzed on the molecular level, randomly chosen to cover all substrates, isotope values, and various localities (Pähl, Fischbachau, Farchant, Gauting, and München). The ITS nrDNA region has been sequenced and analyzed for both bionts separately together with representative sequences from GenBank (Table 2 and 3). MP and ML analysis yielded only one tree each with identical tree topologies, indicating highly reliable results. The sequences of all lichen mycobionts group with high statistical support together with other sequences from *X. parietina* mycobionts obtained from specimens collected in Sweden and Norway by Lindblom and Ekman (2005, 2007), corroborating the morphological determinations (Fig. 4, right-hand side). All photobiont sequences group with high statistical support with the sequence of the authentic strain of *Trebouxia decolorans* Ahmadjian, UTEX 781 (isolated from the lichen *Amandinea punctata* (Hoffm.) Coppins & Scheid. collected in the United States of America). The morphologically similar species *T. aggregata* (P. A. Archibald) G. Gärtner, *T. arboricola* Puym., and *T. crenulata* P. A. Archibald form the highly supported sister group. Thus, all photobionts of the investigated lichen thalli belong to only one algal species, *T. decolorans* (Fig. 4, left-hand side). Differences in the sequences do not show any correlation with the substrate or isotope composition, nor with the geographic origin of the specimen, indicating again that specimens of one and the same species have been analyzed (Fig. 4).

### Isotopic differences between photo- and mycobiont of lichens

**Figure 5:**
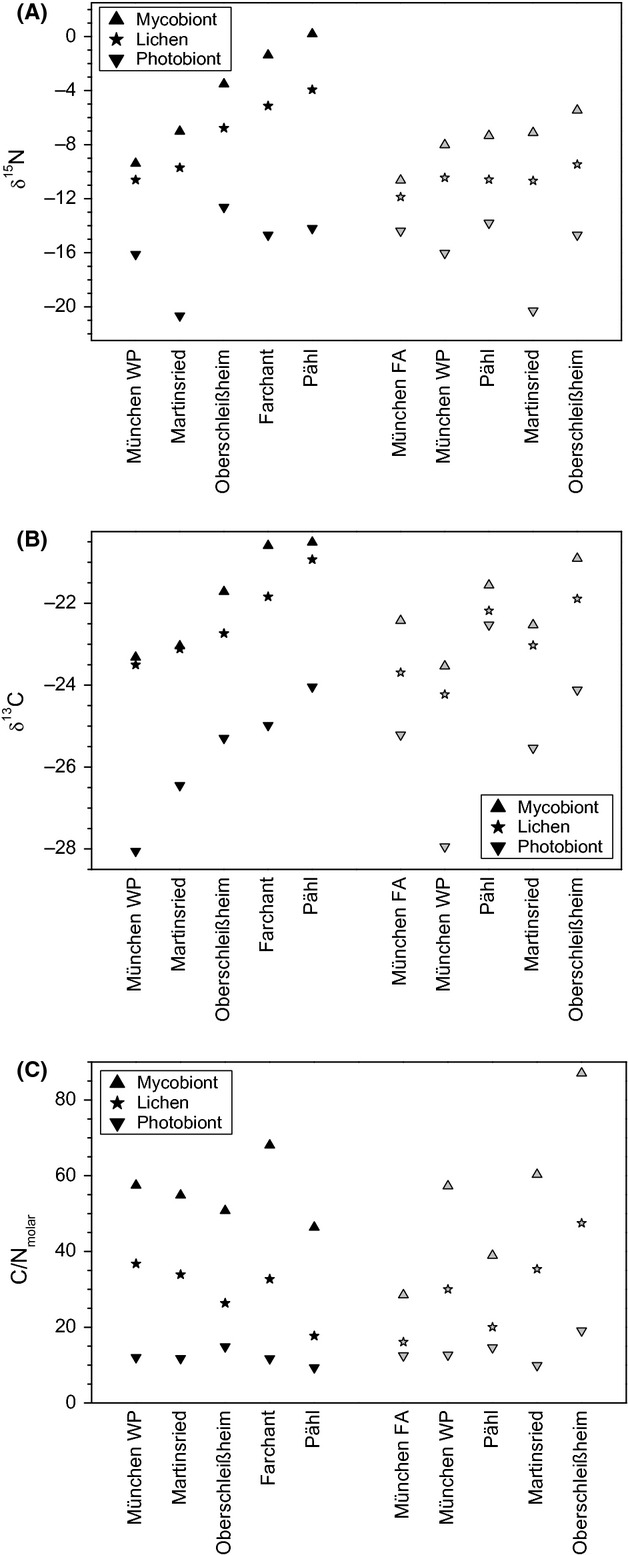
Nitrogen (A) and carbon (B) isotopic composition as well as C/N ratios (C) of 10 *Xanthoria parietina* populations and of myco- and photobionts separated from the same samples. Black symbols represent values from lichens that grew on minerogenic, gray symbols those that grew on organic substrates.

**Figure 6:**
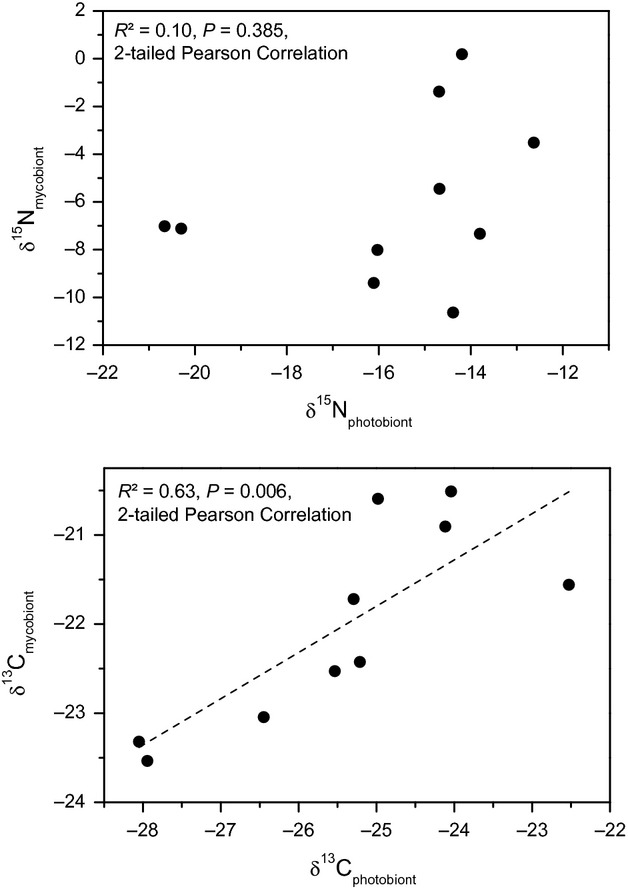
δ^15^N values of the lichens from Figure 5A, photobiont plotted versus mycobiont. δ^13^C values of the lichens from Figure 5B, photobiont plotted versus mycobiont.

Photo- and mycobiont of 10 populations of *X. parietina* were separated from five lichen thalli of each population. Their nitrogen and carbon isotope composition as well as their N content were analyzed. The δ^15^N results show rather little isotopic differences for the *Xanthoria* photobionts with values ranging between −12.6 and −20.7‰ independent of substrate (averages −15.7 ± 3.1‰ and −15.8 ± 2.6‰ for minerogenic and organic substrates, respectively). Mycobionts of *Xanthoria*, however, were always more ^15^N enriched (total range: 0.2 to −10.6‰) and showed larger differences depending on substrate (Fig. 5A). Mycobionts of populations grown on minerogenic substrates had higher δ^15^N values (−4.2 ± 4.0‰) than those grown on tree bark (−7.7 ± 1.9‰). δ^13^C values range from −28.1 to −22.5‰ (mean value −25.4 ± 1.7‰) for photobionts and from −23.5 to −20.5‰ (mean value −22.0 ± 1.1‰) for mycobionts. As for δ^15^N, mycobionts of *Xanthoria* were always relatively enriched in the heavy isotope relative to the photobiont (Fig. 5B). C/N values were lower and less variable for photobionts (mean value 12.9 ± 2.8) than for mycobionts (mean value 55.0 ± 16.0) due to a higher and more stable N content (Fig. 5C). δ^15^N values of photobionts and mycobionts are not correlated, while δ^13^C values are (Fig. 6).

**Figure 7:**
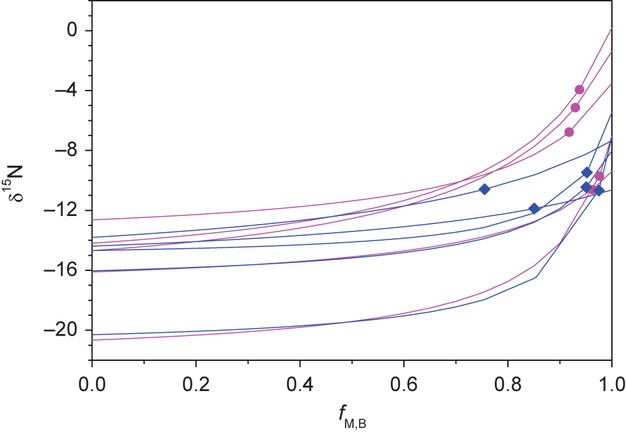
Modelled δ^15^N values of the lichens from Figure 5A with increasing fraction of mycobiont biomass (f_M,B_) based on a concentration-dependent two-end-member mixing model. End points of mixing lines represent δ^15^N values of photo- and mycobionts, respectively. The actually measured δ^15^N values of lichens are given as diamonds on the respective mixing lines (symbols as in Fig. 5A). Colors differentiate lichens grown on minerogenic (magenta dots) and organic (blue diamonds) substrate.

The fraction of mycobiont biomass (f_M,B_) was modelled using equation (6). The modelled nonlinear mixing lines demonstrate that the lichen δ^15^N increases with increasing f_M,B_ (Fig. 7). The f_M,B_ of each sample was determined by fitting the model to the actually measured lichen δ^15^N. The results show that f_M,B_ was similar (90 ± 9%) for the *Xanthoria* selection from organic as for *Xanthoria* from minerogenic substrate (94 ± 2%). Based on the available dataset, the higher δ^15^N means of *Xanthoria* grown on minerogenic compared with organic substrate in Figure 2 can be attributed to (1) higher δ^15^N values of their respective mycobionts and (2) a higher fraction of mycobiont biomass contributing to the total lichen biomass. However, the populations from which the bionts were separated demonstrate that there is an overlap in the isotopic composition as well as in the mycobiont biomass fraction between *Xanthoria* populations grown on different substrates.

## Discussion

### Substrate-specific δ^13^C variability: microclimate or respiratory CO_2_?

The δ^13^C values of *X. parietina* from minerogenic substrate show significantly higher δ^13^C values than those grown on organic substrate (Fig. 3). Inorganic carbon acquisition in lichens is accomplished by the photobiont and, thus, dependent on moisture and light availability (Palmqvist 2000). The observed δ^13^C differences therefore could have been evoked either by different microclimatic conditions on both substrate types or isotope effects due to uptake of isotopically different CO_2_ sources.

Increased δ^13^C values in drier habitats have been found for lichens (Batts et al. 2004). Therefore, the higher water storage capability of organic in contrast with minerogenic substrates is a likely reason for the observed lower δ^13^C values of lichens growing on it. However, very high water contents in lichens (suprasaturation) can increase the CO_2_ diffusion resistance for lichens (Lange et al. 2001). This diffusion limitation decreases the fractionation by 3−4‰ at highest suprasaturation (Máguas et al. 1995) and therefore will result in higher δ^13^C values. Apparently this effect plays a minor role for substrate-related δ^13^C differences investigated in our study.

Besides the water status, the uptake of respiratory CO_2_ can be a second cause for lower δ^13^C values of lichens growing on organic substrates. Lichens growing in the canopy or close to the soil potentially assimilate CO_2_ from plant and soil respiration (Broadmeadow et al. 1992; Máguas et al. 1993, 1995), but also CO_2_ from bark respiration could be taken up by corticolous lichens. This “canopy effect” could shift δ^13^C to more negative values, as the isotopic composition of respired CO_2_ is in the δ^13^C range of organic matter and thus much more negative (−19 to −32‰; Pataki et al. 2003) than atmospheric CO_2_ (−8‰ on average). The more negative δ^13^C values of lichens growing on organic substrate, thus, could be related to respiratory CO_2_. Another source for differences in δ^13^C values in lichen thalli from bark versus rock substrates is additional organic carbon uptake as it is well known that both lichen bionts readily take up organic carbon (Ahmadjian 1965). Whether uptake of respiratory CO_2_, light level, water availability, or additional organic carbon uptake on different substrates, or a combination of these factors, are responsible for the observed δ^13^C differences remains, however, the object of further studies.

### Causes for substrate-specific δ^15^N variability

The N content of lichen thalli is dependent on the nutritional status of the lichens and might be limiting for growth and distribution of lichens (Crittenden et al. 1994; Palmqvist et al. 2002; Nash 2008). When nitrogen is scarce, lichens were found to invest relatively more N in photosynthetically active substances (Chl a and Rubisco) instead of fungal substances (chitin; Palmqvist et al. 1998). At least three possible causes may be responsible for the observed substrate**-**specific differences in δ^15^N values: Preferential selection of ^14^N in short periods of uptake may lead to ^15^N depletion. These N-uptake periods may be temporary due to short-dated wetting (and thus transient metabolic activity) or short periods of nutrient availability. Second, different N sources, like nitrate (liquid) or ammonia (gaseous) may lead to different δ^15^N values due to higher fractionation in the gaseous phase. Third, transfer of organic nitrogen, for example, from the mycobiont to the photobiont may result in ^15^N depletion in a similar way as in mycorrhizal symbiosis (Hobbie and Hobbie 2008), resulting in increased ^15^N depletion of the photobiont and less depletion in the mycobiont. This process would be most likely to occur on bark where organic nitrogen sources in the runoff water are available. Thus, the overall higher δ^15^N values of *X. parietina* that grew on minerogenic substrate may reflect the lower nitrogen availability (Fig. 3). Nitrogen availability and demand determine the ^15^N discrimination in plants (Evans 2001). Lower nitrogen availability, as supposed for the lichens growing on minerogenic substrates, leads to a lower discrimination and hence to higher δ^15^N values of lichen thalli. The same effect as in the lichen *X. parietina* was observed in individual thalli of *Anaptychia ciliaris* and *Phaeophyscia orbicularis* growing on bark or stone, respectively (data not shown). With respect to the N-content of the substrate the stone surfaces contained no measurable N amounts. Measurements for the δ^15^N values of bark ranged from −0.7 to −4.6‰ (mean of −2.7 ± 1.6‰), again providing evidence for isotope discrimination by the lichens given their much more negative values (−9.7 ± 4.2‰ for *Xanthoria* grown on bark). Nevertheless, as lichens are not single organisms, the δ^15^N values of the symbionts need to be taken into account. Our analysis showed that the higher δ^15^N values of *Xanthoria* grown on minerogenic compared with organic substrate can be attributed to (1) higher δ^15^N values of their respective mycobionts and (2) a higher fraction of mycobiont biomass contributing to the total lichen biomass (Figs. 5, 7). A higher photobiont biomass in the specimens with higher N content is well in agreement with data presented by Dahlman et al. (2003) for *Hypogymnia physodes* (L.) Nyl. and *Platismatia glauca* (L.) W. L. Culb. & C. F. Culb.

### Isotopic discrimination between lichen symbionts

δ^15^N values of the photobiont *T. decolorans* are independent from and always more negative than those of the associated mycobiont (Figs. 5A, 6). If the photobiont would gain its N primarily from the mycobiont, one would expect a correlation between these values as the mycobiont N would be the pool for the photobiontal N. This applies to both, active N transport through the fungus to the alga and N leakage from the fungus to the alga. Such a correlation only exists for δ^13^C values (Figs. 5B, 6). A transport of the sugar alcohol ribitol from the *Trebouxia* photobiont to the *Xanthoria* mycobiont is well established (Richardson and Smith 1968; see Eisenreich et al. 2011 for a review) and can easily explain the observed correlation. The independence of the δ^15^N values could be interpreted as further evidence that the *Trebouxia* photobiont is able to compete successfully with the mycobiont for available nitrogen like nitrate as discussed by Dahlman et al. (2004) due to indirect evidence by low nitrate uptake rates in bipartite cyanobacterial lichens as compared to the tripartite lichens, but both with similar nitrogen content. The latter showed a significant higher nitrate assimilation that might have primarily been conducted by the green algal partner *Coccomyxa*. Nevertheless, Pavlova and Maslov (2008) demonstrated the inability of nitrate uptake for the lichenized *Trebouxia* photobiont of *Parmelia sulcata* Taylor. Moreover, δ^15^N values of nitrate in rain in central Europe ranged between +2.4 and −6.5‰ (Freyer 1991) and thus did not reach such negative values as measured in *T. decolorans* in this study (−15.8 ± 2.7‰). Ammonia dissolved in rain (ammonium) is known to be more depleted in ^15^N, ranging from −5.6 to −8.6‰ measured in the United Kingdom (Heaton et al. 1997). Even more negative values are reported for atmospheric ammonia around −10‰ (Erskine et al. 1998; Tozer et al. 2005). The latter authors also demonstrated, that diffusion into the tissue of lithophytes (as simulated by an acidified mat of glass fibers) results in a further ^15^N depletion of 4.0–5.5‰. Thus, the observed photobiontal δ^15^N values between −12.6 and −20.7‰ are very well in accordance with the uptake of atmospheric ammonia as primary N source by *T. decolorans*. This may explain the nitrophilic nature of *X. parietina*, as no bird droppings have been found at the investigated sites. If it is true for lichen algae in general remains to be tested. While atmospheric ammonia may not be the only nitrogen source, the data presented in this study suggest that it is the most important N source for *T. decolorans*. In accordance, ammonia is the most abundant N species in the atmosphere in remote as well as anthropogenic influenced areas (Crittenden 1998; Krupa 2003). Lichen mycobionts, in contrast, can use additional N sources, most notably NH_x_ and NO_x_ species from rainfall as demonstrated by the nitrate uptake of the *P. sulcata* mycobiont (Pavlova and Maslov 2008). The use of these more ^15^N-enriched sources than ammonia is reflected by the more positive δ^15^N values of the *X. parietina* mycobiont. The mycobiont-independent photobiontal δ^15^N values are thus best explained by a mycobiont-independent N uptake of the photobiont of atmospheric ammonia. In *X. parietina*, and possibly in more green-algal lichens, only a very limited (if at all) N transfer seems to occur from the mycobiont to the green-algal photobiont which has been shown for tripartite lichens harboring cyanobacteria and green algae (Rai et al. 1983).
